# “Teaching capital”– a sociological analysis of medical educator portfolios for promotion

**DOI:** 10.1007/s10459-024-10333-3

**Published:** 2024-04-29

**Authors:** Mette Krogh Christensen, I. M. Pedersen, G. Wichmann-Hansen

**Affiliations:** 1 Centre for Educational Development, Aarhus University, Trøjborgvej 82, 8000 Aarhus C, Denmark; 2 Department for Education Studies, Faculty of Arts, Aarhus University, Aarhus, Denmark

**Keywords:** Medical educator, Teaching portfolio, Cultural capital, Higher education, Academic promotion

## Abstract

Medical educator portfolios (MEP) are increasingly recognized as a tool for developing and documenting teaching performance in Health Professions Education. However, there is a need to better understand the complex interplay between institutional guidelines and how teachers decode those guidelines and assign value to teaching merits. To gain a deeper understanding of this dynamic, this study employed a sociological analysis to understand how medical educators aspiring to professorships use MEPs to display their teaching merits and how cultural capital is reflected in these artefacts. We collected 36 medical educator portfolios for promotion from a large research-intensive university and conducted a deductive content analysis using institutional guidelines that distinguished between mandatory (accounting for the total body of teaching conducted) and optional content (arguing for pedagogical choices and evidencing the quality, respectively). Our analysis showed that the portfolios primarily included quantifiable data about teaching *activities*, e.g., numbers of students, topics and classes taught. Notably, they often lacked evidence of quality and scholarship of teaching. Looking at these findings through a Bourdieusian lens revealed that teachers in this social field exchange objectified evidence of hours spent on teaching into teaching capital recognized by their institution. Our findings highlight how institutional guidelines for MEPs construct a pedagogical battlefield, where educators try to decode and exchange the “right” and recognized *teaching capital*. This indicates that MEPs reflect the norms and practices of the academic field more than individual teaching quality.

## Introduction

While *medical educator portfolios* (MEP) have become more prevalent and recognized as a tool for developing and documenting teaching performance in Health Professions Education (Hobson et al., 2022; Stull et al., 2021), there are still many tensions, disagreements, and uncertainties involved in portfolio use (Harrison et al., 2022; Hong et al., 2021). Even so, there has been a striking lack of research focusing on how medical educators and higher education teachers try to decode what is the ‘currency’ of teaching performance, how to accommodate it, and how to document it in an MEP– especially when the MEP is used for promotion in the academic field. In other words, fair judging of teacher portfolios is not only a matter of transparent criteria and sound assessment strategies but also a matter of how these criteria and strategies act as cultural capital (Bourdieu, 1986) which is communicated to and converted by medical educators in an MEP.

The legitimacy of teaching portfolios has been intensively discussed. Advocators of portfolios argue that it is a means to place teaching on an equal footing with academic accomplishments (Hobson et al., 2022), and to support a scholarly and community-based approach to teaching (Pelger & Larsson, 2018). Critics, on the other hand, assert that the teaching portfolio mainly serves as a management tool for monitoring teaching (Buckridge, 2008; FitzPatrick & Spiller, 2010), and that the portfolio is merely an accountability mechanism that universities have introduced in a market-oriented environment in response to the growing quality assurance agenda in HE (Leggett & Bunker, 2006).

Also, the purpose of portfolios has been much debated and analyzed. Early, Smith and Tilemma 2003) offered a useful framework for distinguishing between different dimensions in teaching portfolios: (i) the purpose of the portfolio, as either being selection/promotion oriented or learning/developmentally oriented; (ii) the incentive of the portfolio, as either being mandated by institutional requirements or self-directed/voluntarily initiated for personal use (Smith & Tillema, 2003).

The main challenge is that portfolios are used both summatively and formatively (Buckridge, 2008). On the one hand, portfolios are used for tenure and promotion (Little-Wienert & Mazziotti, 2018) and thus for summative performance appraisal (Hobson et al., 2022; Trevitt et al., 2012). On the other hand, portfolios are used for learning (Pelger & Larsson, 2018; Tofade et al., 2014) and reflection (Hamilton, 2018; Tigelaar et al., 2006) providing a record of continuous learning and growing professionalism (Dalton et al., 2018; Tompkins & Paquette-Frenette, 2010). For example, a study on anaesthetists’ use of teaching portfolios showed that they gain the most benefit from a teaching portfolio when it is used as a tool for self-reflection of their teaching practice and not merely as a summative list of activities and achievements (Sidhu, 2015).

This unsettled multi-purpose of a teaching portfolio tends to produce a range of complex expectations and emotions among educators and academic staff, including even resistance towards writing a portfolio (FitzPatrick & Spiller, 2010; Hamilton, 2018; Trevitt et al., 2012). In FitzPatrick and Spiller’s study (2010), participants were uncertain about whether to structure the text as a private reflection on their individual teaching journey or as a scholarly, analytical text or more as a ‘sales document’ displaying triumphs and achievements. The lack of a clear legitimate purpose and lack of a defined reader may lead to ambivalence, frustration and resistance among academics when trying to write portfolios (De Rijdt et al., 2006).

The highly differentiated purposes– and consequently differentiated structures and content– of a medical educator portfolio are confirmed in a recent systematic scoping review investigating what is known of the definitions, functions, content, implementation and assessment of MEP (Hong et al., 2021). The review was based on a total of 12.360 reviewed abstracts, 768 evaluated full-text articles, and 79 included articles for further thematic analysis. Across the included articles, the authors defined MEP as a portfolio curated by the individual educator, which contains a collection of documents spanning a period of time seeking to demonstrate developing competencies, desirable character traits, learning, challenges and improvements made in the field of medical education. The authors concluded that medical educator portfolios continue to be used for a variety of purposes, roles and goals which remain influenced by “local clinical, academic, personal, research, professional, ethical, psychosocial, emotional, cultural, societal, legal and educational factors” (Hong et al., 2021, p. 12) underlining the heterogeneity of educator portfolios.

Since the structure of a portfolio often varies according to the purpose of the portfolio, defining content or evidence of a good portfolio has been subject to much discussion. Despite varying structures of the teaching portfolio, there appears to be consensus across recent international literature that a ME includes four basic elements (Dalton et al., 2018; Harrison et al., 2022; Little-Wienert & Mazziotti, 2018; Sidhu, 2015; Stull et al., 2021): *Evidence of* quality *of teaching*: student feedback surveys, self-evaluations, peer evaluations, supervisor evaluations, teaching awards, invitations to teach etc.; *Evidence of quantity of teaching*: lists of conducted teaching and supervision, lectures delivered, duties as an examiner etc.; *Philosophy of education*: accounts for approach to teaching, educational strategies and values, pedagogical goals, theoretical foundations etc.; *Scholarship of teaching and learning*: description of involvement in professional development, teaching projects, educational management and leadership, review of relevant educational literature etc.

So far, literature on MEPs has brought important knowledge about the legitimacy, purpose, and structure of the portfolio. However, to the best of our knowledge, sociological analyses of the institutionalized summative use of teaching portfolios are sparse. One example is a study on quality issues in judging teaching portfolios (Tigelaar et al., 2005). Tigelaar and colleagues advocate for the use of a hermeneutic, interpretative approach to the judging of portfolios as well as the use of quality criteria to establish trustworthiness in performing and monitoring portfolio assessment. Consequently, they proposed a constructivist approach aimed at generating maximum diversity in interpretations by the different stakeholders. Although time-consuming and resource-intensive, these recommendations seem inevitable and sound to ensure quality in using MEP as a tool for promotion. Accordingly, research is needed to better understand the logic of MEP for promotion and how teaching portfolios function as an exchange of specific forms of cultural capital between staff members and the universities.

### Aim of the study

Using Bourdieu’s theoretical framework of social field– in particular, the concept of *cultural capital* which will be outlined in the next section of the paper– this study investigates how medical educators aspiring to a professorship within the social field of Health Professions Educations use the teaching portfolio to outline their teaching merits. A recent study has demonstrated that Bourdieu’s idea of social fields as competitive arenas that possess an internal logic and specific sets of appreciations and assignment of privileges, e.g., grants, positions, and salary, is useful to make sense of institutionalized summative uses of teaching portfolios (Weinreb & Yemini, 2023). In this article, we zoom in on how teaching portfolios are a token of different forms of cultural capital. Bourdieu’s concept of capital roughly means power or resources; it comes in several forms that under certain circumstances can be transformed into one another (Granovetter & Swedberg, 2011). Therefore, we base the study on the assumption that teaching portfolios involve an *exchange of cultural capital* between the institution and the teacher. On the one hand, educational institutions (including medical schools) increasingly *value* (or demand) that teachers maintain and hand in teaching portfolios. On the other hand, medical educators construct teaching portfolios as a form of *valuation* of their teaching performance.

In this paper, we assume that the written teaching portfolio handed in for promotion is a representation of a specific form of cultural capital, namely *‘teaching capital’*, which differs from other forms of capital such as *‘research capital’*, i.e., publication lists, funding, academic achievements, and *‘clinical capital’*, i.e., quantity and quality of surgery and treatments, technical skills, communication skills etc. Hence, for a start, we identify teaching capital as the individual staff member’s total set of cultural capital that can be recognized as legitimate teaching competence. It is a *currency* that is always subject to power negotiations in the exchange of cultural capital. Thereby we aim to challenge and expand the field of research on medical educator portfolios for promotion by viewing the teaching portfolios of newly employed or reemployed Professors as forms of cultural capital that function as implicit and explicit references in judging the portfolio for promotion. The question driving our research is:

Which forms of cultural capital are at stake in the exchange of teaching portfolios for promotion? And how is teaching capital constructed through teaching portfolios?

Below we account for the concept of *cultural capital* by reference to Bourdieu’s theoretical framework.

### Cultural capital– a theoretical framework

We base the study on reflexive sociological analysis of Professors’ teaching portfolios. More specifically, we introduce sociologist Pierre Bourdieu’s concept of *cultural capital* (Bourdieu, 1986, 1998) as a theoretical framework for understanding the function of teaching portfolios in research-intensive universities. This concept provides a useful lens for investigating norms, practices and ‘drivers’ along with other sociocultural aspects of Professors’ educational merits.

It is impossible to account for the structure and functioning of the social world– and of education, in particular– unless one reintroduces capital in all its forms and not solely in the one form recognized by economic theory. Bourdieu’s concept of capital is based on his theory of power relations and his attempt to understand how power, including the power of social norms, works without explicit coercion (Grenfell, 2014). Bourdieu understood the social world as a multidimensional social space consisting of multiple social fields in which interests, conflict and competition appear simultaneously (Bourdieu & Nice, 2004). His understanding of society can be illustrated by metaphors of ‘a game’ with rules and a playing field where certain forms of capital are more valued than others. In this paper, we regard Health Professions Education (HPE) as an academic field in which the main interests are research (publications, funding of research projects represented in the research CV) and education (teaching students, assessment, supervision, etc.). A social field such as HPE shares structures such as institutional objects and regulations. Although individual agents occupy either orthodox or heterodox positions within the social field, their positions are clustered and distributed by the *homology* of the field, that is, the structural (often hidden) similarities across (often visible) differences within the field.

According to Bourdieu, human practice involves an element of symbolic value which is ascribed to certain forms of *cultural capital*, realized or not by the individual person:

Capital is accumulated labor (in its materialized form or its “incorporated,” embodied form) that, when appropriated on a private, that is, exclusive, basis by agents or groups of agents, enables them to appropriate social energy in the form of reified or living labor (Bourdieu, 1986, p.241).

Capital is a force and a resource inscribed in objective or subjective structures, for example in hierarchies of academic ranks and in selection criteria in promotion systems, but it is also the principle underlying the immanent regularities of the social world, for example the norms and tacit knowledge that regulate academics’ priorities and balance between research and teaching, their views on career opportunities and promotions in academia.

It is what makes the games of society—not least, the economic game—something other than simple games of chance offering at every moment the possibility of a miracle (Bourdieu, 1986, p.241).

In other words, cultural capital does not operate by chance, but by human actions.

## Methods

The study was designed as a document analysis, which includes finding, selecting, analyzing, synthesising and interpreting data contained in documents (Bowen, 2009). Document analysis has the potential to disclose the developments of norms and practices within organizations as well as a representation of the rationale of the document at the time it was written (Lynggaard, 2022). In the context of HPE research, document analysis is most often used for contextual and triangulation purposes (Cleland et al., 2023). However, the choice of document analysis as the primary (and sole) method in this study reflects our understanding of the MEP as a *boundary object*, that is, a document that in its own right may serve to connect and mediate between multiple social worlds (Cleland et al., 2023, p. 412) and translate concerns and intentions across culturally defined boundaries (Fox, 2011). Therefore, this research method was chosen because it serves the purpose of our study, which was to systematically describe and analyze how the participants display their educational merits to meet the institutional and cultural norms.

### Participants and document sample

Our sample consists of 36 teaching portfolios collected during the spring/summer 2022 from male and female Professors at the Faculty of Health at a large research-intensive Danish university. In Denmark, a full Professor is a university teacher and researcher (and sometimes also a clinician) of the highest academic rank. Apart from their research and clinical work, Professors in this study context are responsible for planning and conducting teaching of bachelor and master’s students as well as graduate students, and for supervising younger teachers and clinicians. Teaching is therefore an integrated part of the job as a full Professor.

**Fig. 1:**
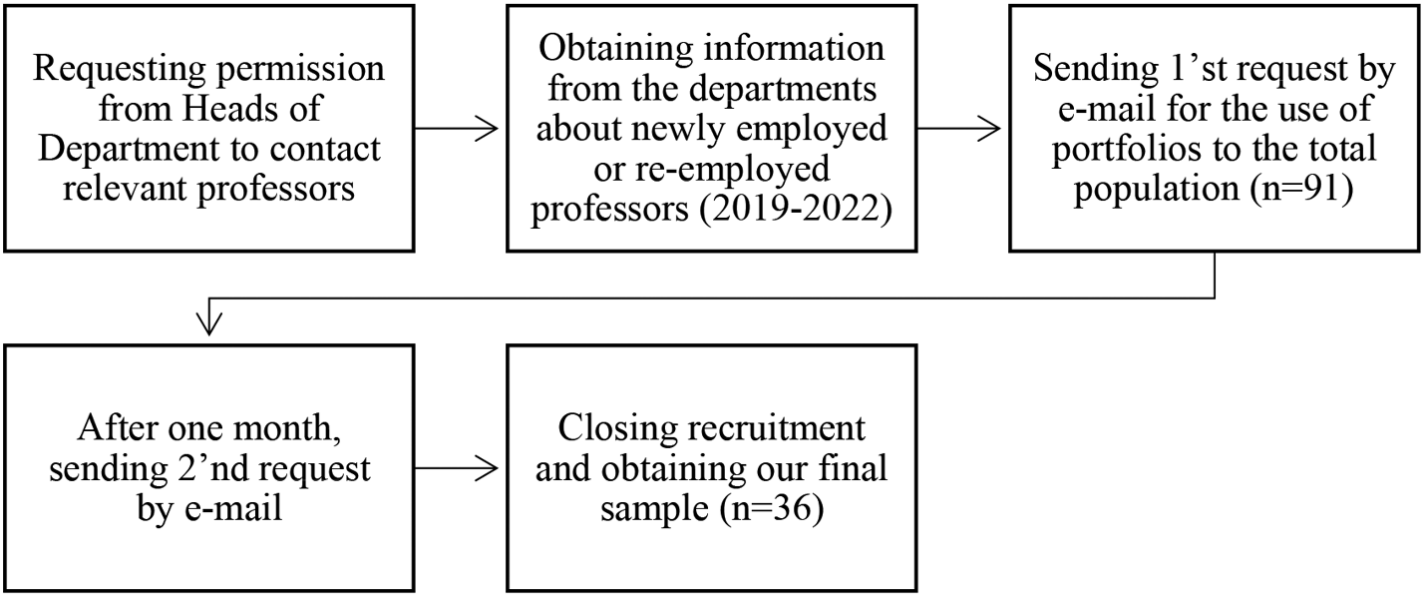
Flowchart of recruitment process

In the Danish Higher Education context, it has recently become mandatory to append a teaching portfolio when applying for academic tenure and promotion, including applications for full professorships. Following permission from the Faculties’ Heads of Department, we enquired the total population of Professors at the Faculty (*n* = 91) by email about sharing their teaching portfolios used in the application process for their new employment or reemployment as Professors in the period of 2019–2022. After sending 1st and 2nd request by e-mail to the 91 Professors (see Fig. 1), we received 36 portfolios.

Compared to the institution’s public website about the scientific staff in the included departments, the sample was considered to be representative of the general population at the Faculty of Health in terms of departments, academic backgrounds, educational programs, and gender distribution.

### Data analysis

We undertook a content analysis using a deductive approach with the intension of producing a quantitative overview of the material as the basis for conducting a theoretical interpretation (Fereday & Muir-Cochrane, 2006). The analysis followed two steps. Firstly, we succumbed the portfolios to a deductive coding strategy using the institutional portfolio guideline in the study context as an a priori template for coding the data material (see Table 1). The institutional guideline suggests that a teaching portfolio includes three sections: (1) a required section of description and documentation of teaching, (2) an optional section for reasons for choices in relation to teaching, and (3) an optional section for results, e.g., evaluations documenting the quality of teaching.

**Table 1:** A priori thematic framework based on institutional guidelines

Themes in the institutional guidelines	Subthemes	Content of subthemes
1. Description and documentation of teaching (Required)	1.1. Conducted teaching	Role, scope, type, and level of conducted undergraduate teaching (university level).
	1.2. Course management	Experience with course management or course responsibility, including arranging symposia.
	1.3. Examinations carried out	Role, scope, type, and level of examinations carried out.
	1.4. Supervision	Role, scope, type, and level of supervision, e.g., Master’s thesis, PhD and similar tasks, e.g., PhD assessment committee member etc.
	1.5. Pedagogical merits	Information and documentation relating to completed courses in university pedagogics or other pedagogical courses including communicative training.
	1.6. Collaboration about teaching	Experience working in teams of teachers, collegial supervision etc.
	1.7. Postgraduate teaching and degree program management	Experience with degree program management and development, including postgraduate teaching and further and continuing education.
	1.8. Development of subject areas	Contribution to the development of subject areas, subjects, or disciplines.
	1.9. Textbooks or teaching material	Contribution to textbooks or teaching material.
	1.10. Other teaching*	Other experience in the area of teaching and university pedagogics including teaching outside the university and medical education, e.g., “talks” aimed at patient groups, the public/laymen, administrators and other learners, experience with press contact.
2. Reasons for choices in relation to teaching (Optional)	2.1. Teaching objectives and–strategies	Description and reflection about teaching objectives and–strategies and forms of examination.
	2.2. The teacher’s role and the role of the students	Description and reflection about the teacher’s role and the role of the students.
	2.3. The applicant’s own pedagogical development	Description and reflection about the applicant’s own pedagogical development.
3. Results (Optional)	3.1. Student evaluations	Documented evaluations from students.
	3.2. References	References from, for example, the director of studies, course manager, the head of the department or others in connection with pedagogical development.
	3.3. Other indicators of teaching quality	Other indicators of teaching quality, e.g., level of activity, completion rates, absence, average marks, and anecdotal descriptions.

*All participants account for the dissemination of research at subject-specific conferences. As these accounts mirror a research CV, they are not included in subtheme 1.10. “Other teaching”

The three sections in the institutional guidelines correspond very well with general guidelines suggested in the international literature on teaching portfolios, which justifies our use of a single institutional practice as a general analytical template. During the coding process, however, we added one extra theme labelled “other”, because some of the portfolios included material that exceeded the three sections in the guideline. Secondly, we undertook a theoretical reading of the resulting quantitative overview of the material applying the Bourdieusian concept of cultural capital to ascertain the teaching capital represented by the portfolios.

### Reflexivity

Reflexivity is a shared and cooperative research practice that involves self-conscious critique and appraisal in the research team (Olmos-Vega et al., 2022). The team conducting this study was composed by experienced researchers within faculty development in medical education (MKC) and teaching and learning in higher education (GWH and IMP), who have worked together as researchers and faculty developers for years and thus have had “time to build rapport and grapple with decisions and data together” (Olmos-Vega et al., 2022, p. 249). The cross-disciplinary quality of the group strengthened the discussions and decisions during the research process. First, IMP collected the portfolios and made the initial deductive coding of data. MKC and GWH acted as sparring partners in this first phase of the study. Then, all three researchers critically examined the findings and finalized the resulting quantitative overview. MKC conducted the initial theoretical interpretation of the data, and lastly, all three authors constructively finalized the manuscript.

### Compliance with ethical standards

The study was carried out in accordance with responsible conduct of research and was registered accordingly. To ensure organizational transparency and -legitimacy, we gained permission from the Heads of Department before contacting the Professors with the request to use their teaching portfolios for research. We obtained informed consent from all individual participants included in this study. After receiving extensive information about the project and the processing of personal data, all participants voluntarily made their teaching portfolio available for the study. Participants were recommended to remove sensitive personal data before sending their portfolio to us.

This is an observational study. The Aarhus University Research Ethics Committee has confirmed that no ethical approval is required. Data was approved by the Data Protection Unit (file number 2016-051-000001, sequential number 2622) and processed in accordance with the General Data Protection Regulation (GDPR) article 6.1.e (general personal data).

### Findings

The coding of the included teaching portfolios revealed an uneven distribution of the three themes: (1) description and documentation of teaching, (2) reasons for choices in relation to teaching, and (3) results, e.g., evaluations documenting the quality of teaching.

As shown in Table 2, all portfolios contained some sort of *descriptions and documentation of teaching* (undergraduate and postgraduate), supervision, examinations carried out, contribution to textbooks or other teaching material, and experiences with degree program management or course management. These teaching activities were most often listed in tables including year, title of the course or supervised student, type of teaching, hours of teaching, number of students, role of the teacher, level taught, type of exam and/or type of textbook or teaching material. These tables demonstrated the *quantity* of teaching in terms of how many hours, students, topics, and academic levels the individual teacher has taught and/or supervised, and thus comprised the “raw” data depicting the teaching experiences of the applicant.

In this study context, it is an established norm that Assistant Professors complete a course in university pedagogics prior to the appointment as Associate Professor. Thus, it was surprising that one out of five participants in this study had not completed such a course or other pedagogical courses prior to their application for the position as Professor at the university (subtheme 1.5. in Table 2).

We found only sparse evidence of scholarship of teaching (theme 2) and quality of teaching (theme 3) in this sample of teaching portfolios for promotion. Half of the portfolios in our sample included written reflections about reasons for choices in relation to teaching (theme 2 in Table 2), but only one out of five participants included reflective texts about own pedagogical development (subtheme 2.3. in Table 2) or their view on the roles of teachers and students in university (subtheme 2.2. in Table 2). Similarly, only two out of five portfolios encompassed evidence of the quality of teaching in terms of documented evaluations from students, references, or other indicators of quality of teaching (theme 3). The evaluation data for teaching varied from uncommented copies of quantitative standardized student evaluations to reflexive text about how student evaluations had contributed to changes in the teaching. Only five of the 36 portfolios featured letters of recommendation, peer evaluations, or other documents provided by external partners such as peers and educational leaders (see subtheme 3.2. in Table 2).

**Table 2:** Distribution of themes. Number of teaching portfolios coded in the three themes and the 16 subthemes

Themes and subthemes sorted by number of portfolios	Number of portfolios coded in the themes and subthemes	% of total number of portfolios
*1. Description and documentation of teaching (Required)*	36	*100*
Conducted teaching (1.1.)	36	*100*
Postgraduate teaching and degree program management (1.7.)	30	*83*
Supervision (1.4.)	30	*83*
Examinations carried out (1.3.)	29	*81*
Pedagogical merits (1.5.)	28	*78*
Course management (1.2.)	25	*69*
Textbooks or teaching material (1.9.)	20	*56*
Development of subject areas (1.8.)	13	*36*
Other teaching (1.10.)	11	*31*
Experience working in teams of teachers, collegial supervision etc. (1.6.)	10	*28*
*2. Reasons for choices in relation to teaching (optional)*	18	*50*
Teaching objectives and–strategies (2.1.)	18	*50*
The teacher’s role and the role of the students (2.2.)	8	*22*
The applicant’s own pedagogical development (2.3.)	8	*22*
*3. Results (optional)*	15	*42*
Student evaluations (3.1.)	9	*25*
Other indicators of teaching quality (3.3.)	7	*19*
References (3.2.)	5	*14*
Number of portfolios including both optional themes: 2. Reasons and 3. RESULTS	20	*55*

## Discussion

Overall, the finding from the document analysis depicts a population of medical educators with a diverse approach to the portfolio genre in general, and a highly diverse approach to the institution-specific portfolio guidelines. In the next section, we will discuss this finding by applying a Bourdieusian reading of the data to better understand the logic behind the use of MEP for promotion.

Let’s start the discussion with a quotation from Bourdieu on the assumed connection between a scientist and a scientific field– in our case: a medical educator and an academic field:

A scientist is a scientific field made flesh, an agent whose cognitive structures are homologous with the structure of the field and, as a consequence, constantly adjusted to the expectations inscribed in the field (Bourdieu, 2004, p.41).

This quotation illustrates a fundamental premise in Bourdieu’s theoretical apparatus: the homology in the way in which orthodox and heterodox positions are clustered and distributed across a social field such as the academic field. This premise is key to understanding how teaching portfolios function as an exchange of specific forms of cultural capital between staff members and the universities. Based on document analysis of 36 teaching portfolios handed in for promotion, this study set out to explore which forms of cultural capital are at stake in the exchange of teaching portfolio for promotion, and how teaching capital is constructed through the teaching portfolios for promotion. We found that only half of the portfolios included only the required content while the other half included both required and optional content. Seen from a Bourdieusian perspective, this finding could be interpreted as the Professors’ orthodox understanding of the institutional portfolio guideline: they only need to include evidence that is required, because it represents the teaching capital which is recognized by the institution. Accordingly, optional content is misrecognized teaching capital. The other half of the teaching portfolios assume a more heterodox position in this context because they included both required and optional content (theme 2 and theme 3 in Table 2) even though the institutional guideline explicitly indicate that the optional part is not required to obtain the Professor position. At stake here is a central battle over “doxa” (Bourdieu, 1998), that is a battle over the norms, standards, and assumptions in the academic field of Health Professions Education and over the “distinctions between what is good and what is bad” (Bourdieu, 1998, p. 8) in relation to teaching capital in the academic field. In the following, we will further discuss the implications of this finding.

### From embodied to institutionalized cultural capital

Using a Bourdieusian prism in the interpretation of the above finding, we will argue that the sample of MEPs in this study exposes the transformation of embodied and objectified states of cultural capital into an institutionalized state of cultural capital, which is validated by the university and thus converted into a capital of qualification.

The embodied state presupposes a body and a process of incorporation. Embodied cultural capital implies physical labor of assimilation. It costs time which must be invested personally by the investor (in this case, the teacher), whereas institutionalized cultural capital is the objectification of cultural capital in the form of a certificate, a diploma or an academic title (in this case, the professorship) which “confers on its holder a conventional, constant, legally guaranteed value with respect to culture” (Bourdieu, 1986, in: Granovetter & Swedberg, 2011, p. 83). All the teaching, lecturing and supervision the teachers have done (with their bodies), the hours they were present in the classroom and the lecture hall– all the accumulated physical labor– is something that only an individual bodily investment can bring about. *The teaching body in the classroom* may well be the underlying proof of cultural capital in the education system, so to speak. As such, the required tables in the MEPs displaying the quantity of teaching are a representation of the individual teacher’s accumulated amount of cultural capital in the embodied state.

Since the institutional portfolio guideline provided by the university in this study context only required a quantitative account of conducted teaching, it appears meaningful that (some) teachers make a direct and immediate conversion between, on the one hand, accumulated embodied cultural capital in the form of the their own personal investment in hours spent on teaching, and, on the other hand, the cultural capital that is recognized by the institution as teaching merits when applying for an academic promotion. As shown in Table 2, the MEPs in our sample accounted for different types of cultural capital, but all teachers accounted specifically for the embodied state of cultural capital being the actual amount and extent of teaching. Indeed, 50% of our sample used merely an ‘embodied currency’ in their MEP, and yet they were awarded the professor title. Based on these observations, we suggest that *teaching capital* in medical education is first and foremost constructed as the actual and quantifiable number of hours, students, topics, and academic levels the individual teacher has taught and/or supervised.

### Scholarship of teaching: objectified cultural capital

The above suggested definition of teaching capital leaves us with the question: how should we interpret the important finding that in fact 50% of our sample included *both* required and optional content? To answer this question, we turned to the third form of cultural capital: the objectified form. The objectified state of cultural capital is the materialization or realization of objects that exists only because of “the relationship with cultural capital in its embodied form” (Bourdieu, 1986, in Grenovetter & Swedberg, 2011, p. 82), that is, one must have access to the embodied state of cultural capital in order to appropriate and use the relevant objects in accordance with their specific purpose in the specific social field. Consequently, in this study, we regard artefacts such as text products (e.g., written reflections on own teaching experiences or own pedagogical competences/development), scholarly texts (e.g., review of relevant educational literature, accounts for teaching philosophy or educational strategies), and other objectified evidence of quality of teaching (e.g., student feedback surveys, self-evaluations, peer evaluations, supervisor evaluations or letters of recommendation) as an objectified state of cultural capital. This kind of capital can be appropriated and used by the teacher only if the teacher engages with the objects in a purposeful way, and if the teacher is actually involved in the educational setting from which the objects emanate. Taken together, the objectified state of cultural capital presented here resembles *scholarship of teaching* (Fincher et al., 2000) as described by Fincher et al.:

Teaching becomes scholarship when it demonstrates current knowledge of the field and current findings about teaching, invites peer review, and involves exploration of students’ learning […] Teaching in various venues […] can be scholarly if appropriate evidence is presented to show that defined standards have been met. Other learning-related activities […] also can be scholarly if appropriate evidence is presented(Fincher et al., 2000, p. 888).

Recently, a growing interest and recognition of scholarship of teaching in medical education (Irby & O’Sullivan, 2018; Jamieson, 2023; Steinert et al., 2019) is advocated as a distinct quality in the development of medical education. Accordingly, there appears consensus across recent literature on quality of teaching and standards for teaching portfolios in higher education that a teaching portfolio should include four basic elements (Harrison et al., 2022; Hunt & Chalmers, 2021; Little-Wienert & Mazziotti, 2018; Sidhu, 2015; Stull et al., 2021).

1. Evidence of quality of teaching (student feedback surveys, self-evaluations, peer evaluations, supervisor evaluations, teaching awards, invitations to teach etc.).
2. Evidence of quantity of teaching (lists of conducted teaching and supervision).
3. Teaching pedagogy (accounts for educational strategies, goals, and objectives).
4. Scholarship of teaching and learning (description of involvement in professional development, teaching projects, review of relevant educational literature, etc.)

This trend may have inspired some of the teachers in our study to establish their scholarship of teaching and to include evidence of quality of teaching, teaching pedagogy and/or SoTL in the teaching portfolio for promotion.

From a Bourdieusian perspective, it is likely that these teachers include the objectified state of cultural capital in their teaching portfolio for promotion in order to distinguish themselves from orthodox positions in the field and challenge the homology that takes place between positions across a social field and the cultural practices among the agents in the field (Wang, 2016). In this way, they expand their cultural capital to include *a variety of currencies* (embodied as well as objectified capital) and thus claim a heterodox position. Since the teachers are already recognized agents in the academic field of Health Professions, it is most likely that they already know that the expectations inscribed in the field of research-intensive universities is to set high standards for research. They have also been socialized in an academic system where the dominant position is that of the researcher, while teaching has lower status (Weenink et al., 2023), and yet they chose to occupy a less doxic (cf. doxa) position. This main finding in our study may be evidence of tensions in the field where the cultural production and reproduction of positions and practices emerge from the exchange of (sometimes very) different forms of capital. Indeed, most often new trends and dynamics in a field start as strong oppositional voices which gradually may “crystallize into stabilized schemes, meanings and patterns” (Wang, 2016, p. 358).

### Teaching portfolios for promotion display more about the traits of the institution than its teachers

By separating the quantity of teaching (required content) and the scholarship of teaching (optional content), the institution constructs a pedagogical battlefield (Beattie, 2018) where teachers need to read between the lines and to own the insiders’ doxic knowledge to consider whether or not to include the optional content. When teachers’ scholarly reflections on their educational practices, including documentation of the quality of their teaching, is reduced to optional parts of the MEP, it follows that precisely this form of capital is misrecognized by the appointing institution and thus not by default considered transformable into an institutionalized cultural capital. Yet, from a progressive education point of view, accounts of the dynamic formation of a teacher’s professional habitus (Steinert et al., 2019) and the exercise of scholarship of teaching and learning (Felten, 2013) could be part of the teaching capital. Symbolic fields such as the health professions educations (Paton et al., 2021) establish hierarchies of discrimination where some things are better or more worthy than others (Moore, 2014), and some portfolio content is better or more worthy than other content.

Following this logic, we argue that our findings challenge the *social alchemy* of this particular academic field, because many of the teachers in our sample insisted on having their scholarship of teaching recognized in the process of acquiring institutionalized cultural capital. According to Bourdieu, the social alchemy includes cultural norms and socially established practices expressed in institutional documents or artefacts which then “produces a form of cultural capital that has a relative autonomy vis-à-vis its bearer” (Bourdieu, 1986, in: Grenovetter & Swedberg, 2011, p. 83). Or in other words, social alchemy is the institutionalized *common sense* that in the case of the separation of required and optional content in teaching portfolios institutes the norm of quantity (required content) through optionalising scholarship of teaching (optional content). Hence, it follows that the social alchemy of the studied context urges the teachers to think of their pedagogical competences in a particular way that smoothly and directly converts quantifiable teaching labor into an institutionalized state of capital in the form of a certificate of cultural competence (the teaching portfolio) without the intermediate conversion via the objectified state of cultural capital.

It is clear from our findings that the institutional portfolio guideline functions as social alchemy that produces field-specific norms, which then lead to some (and not other) definitions of valued teaching competences, some (and not other) strategies for pedagogical competence development, the prioritization of some (and not other) types of scholarship of teaching and learning, and the appraisal of some (and not other) substantiations of teaching experiences. These distinctions between what is recognized and not recognized as institutionalized cultural capital creates a reality that the teachers try to assimilate. Therefore, the teaching portfolios handed in for promotion reveal more about the traits of the institution than its teachers.

In the case of this study, we conclude that, up till now, *how much* one teaches is bestowed with more cultural capital than *how and why* one teaches. The exchange of cultural capital in the process of writing and not least evaluating MEPs is dynamic, time consuming and resource-intensive (Tigelaar et al., 2005). However, as shown in this study, when agents (medical educators applying for professorships as well as the assessment committees) capitalize on the possibilities to advance the status of scholarship of teaching and succeed in converting it into an institutionalized form of cultural capital in an MEP, then perhaps quality of teaching will gain more recognition in the promotion processes in research-intensive universities?

### Limitations

This study has some limitations. To preserve anonymity of the participating Professors, affiliation, gender, and age are not included as variables for analysis. These variables could have provided more depth and context to the results, which in turn could have enabled a broader understanding of the concept of teaching capital and enhanced the readers’ ability to make analytical generalizations (Kvale & Brinkmann, 2009). However, the relatively high number of participating professors representing a broad spectrum of educational programs provides a general representation to support the findings.

Another limitation relates to the sole deductive analytical strategy focusing on producing a quantitative overview as the basis for theoretical interpretation. Triangulating this strategy with an inductive approach to document analysis could have benefitted the study as it would have enabled a qualitative exploration of Professors’ reasons for choices in relation to teaching to further nuance the concept of teaching capital and qualify discussions of organizational measures applicable to enhance a social alchemy that ameliorates quality teaching. Instead, we conducted a sociological reading of the quantitative overview of the data material applying the Bourdieusian concept of cultural capital to gain a deeper understanding of the dynamic interplay between higher education institutions and how medical educators assign value to teaching merits.

### Conclusions and implications

Our study contributes to research on MEPs in two significant ways. Firstly, we found a predominant focus on quantitative evidence of teaching in the portfolios, with limited emphasis on reflective practices and indicators of teaching quality. This disparity was analyzed and understood by means of the theoretical concept cultural capital. The analysis revealed that the ambiguous nature of institutional guidelines for MEPs are pivotal because teachers who are writing portfolios for promotion are understandably trying to decode how to exchange their merits into currency valued by the institution. In this study context, the institutional guidelines prioritize the quantity of teaching over the quality by making the first part mandatory. It creates a pedagogical battlefield, where teachers need to navigate between required and optional content, representing tacit institutional norms for recognized teaching capital. Furthermore, this battlefield gives precedence to those teachers who have access to the insiders’ doxic knowledge about how to reproduce or break with traditional expectations. As a result, MEPs might end up reflecting the norms and practices of the academic field more than individual teaching quality.

Secondly, our study is pioneering by applying Bourdieu’s theoretical framework in a document analysis of MEPs. By using the concept of cultural capital, we were offered a valuable systematic lens for comprehending how institutional teaching norms and preferences are both generated and manifested within teaching portfolios. Without this lens, there was a risk that we had brushed aside or misinterpreted the many quantifiable lists of conducted teaching hours as rigid, unqualified, or unreflective teachers. Instead, it enabled an analysis of the more complex interplay between institutional guidelines and how teachers can position themselves as adhering to or widening expectations.

### Implications

Decisionmakers in health professions education may use the insights in this study as a stark reminder of how surprisingly differently the MEP is interpreted by teachers within the same institution as a genre and as a document for promotion. We propose that stakeholders in higher education institutions must be mindful of the tremendous influence their guidelines have on determining the value attributed to teaching. Concretely, we suggest that guidelines for writing a teaching portfolio for promotion should be (1) unequivocal, that is, the distinction between required and optional content should be avoided, and (2) based on internationally acknowledged research and standards for teaching portfolios.

Being faculty developers ourselves, we (the authors of this paper) are often asked by staff members why and how to compose a teaching portfolio for promotion. This study has sharpened our understanding of the delicate situation of medical teachers trying to ‘play the game’ of academia and converting scholarship of teaching and learning into an acknowledged form of capital. Indeed, we became more aware of the politicized aspect of our role as faculty developers in the intersection between institutional portfolio guidelines and our own pedagogical dispositions. We encourage colleagues to use our study as a launchpad to critically evaluate their own contribution as faculty developers to the discourse on teaching portfolios for promotion in their respective contexts.

Finally, this study has theoretical implications as it suggests the value of applying sociological analyzes in future studies to further explore how a teaching portfolio functions as a representation of the institution’s doxa or put differently: as a gatekeeper for the institution. A constructive redirection of future research could focus on how teaching portfolios are assessed by external as well as internal assessment committees. It calls for a discussion about to what extent a reflective medical educator teaching portfolio is valued as a full-fledged part of upcoming Professors’ cultural capital in line with a publication list or a track record of funding.
